# Supplementary material for: SUMOylation regulates Lem2 function in centromere clustering and silencing
Source: J Cell Sci. 2023 Dec 1;136(23):jcs260868. doi: 10.1242/jcs.260868 (PMC10730020; doi:10.1242/jcs.260868)
Supplement: Supplementary information [file joces-136-260868-s1.pdf]

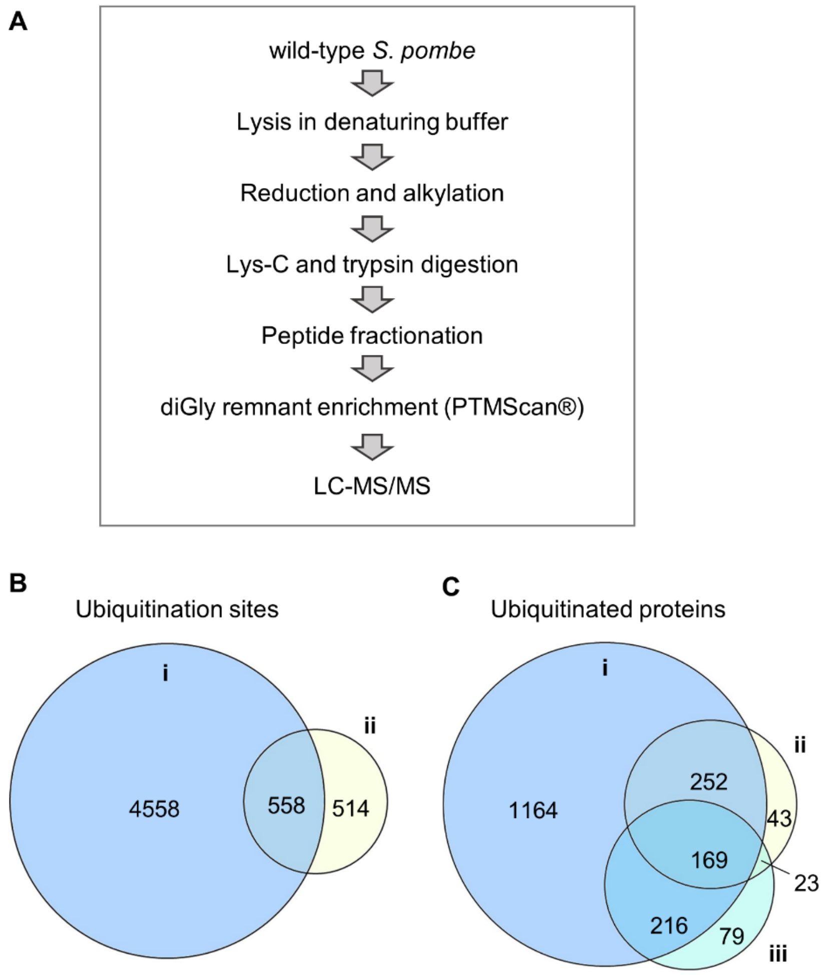

Fig. S1. Global analysis of ubiquitination sites in fission yeast. (A) Outline of the approach employed to identify ubiquitination sites under physiological conditions. **(B)** Comparison of the number of ubiquitination sites identified in this study (i) versus the previous study (Beckley et al., 2015) (ii). 558 sites identified previously were found in this analysis, equating to ~55% of the total ubiquitination sites previously identified. **(C)** Comparison of the number of ubiquitinated proteins identified in this study (i) versus the previous study (Beckley et al., 2015). The latter are divided into those identified directly by identification of diGly peptides (ii); and those identified indirectly based on altered abundance in the absence of deubiquitinating enzymes (iii). 169 proteins were identified in all three analyses.

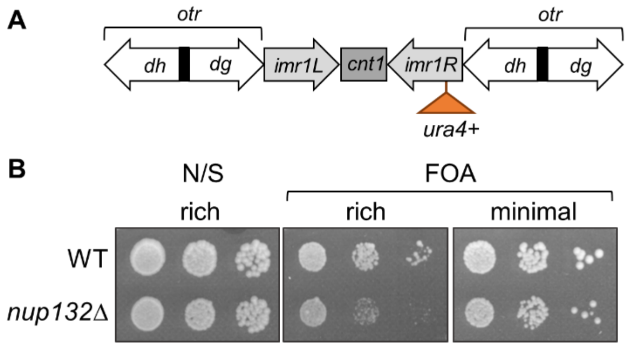

Fig. S2. Deletion of *nup132*+ results in defects in centromeric silencing in rich media but not minimal media. (A) Schematic representation of the *imr1:ura4*+ reporter, indicating the position of the *ura4*+ insertion in centromere one relative to centromeric outer repeats (*otr*; *dg* and *dh*), innermost repeats (*imr*) and central core (*cnt*). **(B)** Assay for silencing of the *imr1:ura4*+ reporter in rich (YES) or minimal (PMG) media: loss of silencing results in increased expression of *ura4*+ and therefore decreased growth in the presence of the counter-selective drug 5-FOA. Growth in the absence of 5-FOA (non-selective, N/S) is shown as a control.

Journal of Cell Science • Supplementary information

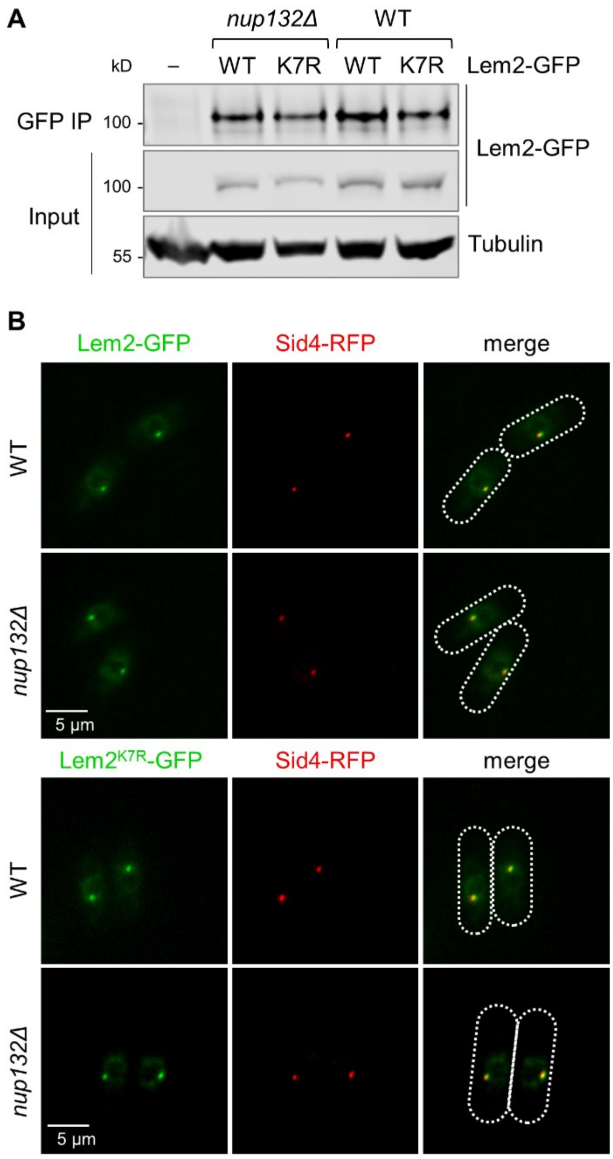

Fig. S3. Lem2 stability and localisation are unaffected by mutation of Lem2 SUMOylation sites or deletion of *nup132*⁺. (A) Western blot analysis of Lem2-GFP, or Lem2^{K7R}-GFP, immunoprecipitated from wild-type or *nup132*Δ cells. Tubulin (anti-Tat1) serves as a loading control. **(B)** Representative images from two-colour live cell imaging of Lem2-GFP, or Lem2^{K7R}-GFP, and Sid4-RFP (SPB marker) in wild-type and *nup132*Δ cells. Dotted lines indicate cell boundaries.

Journal of Cell Science • Supplementary information

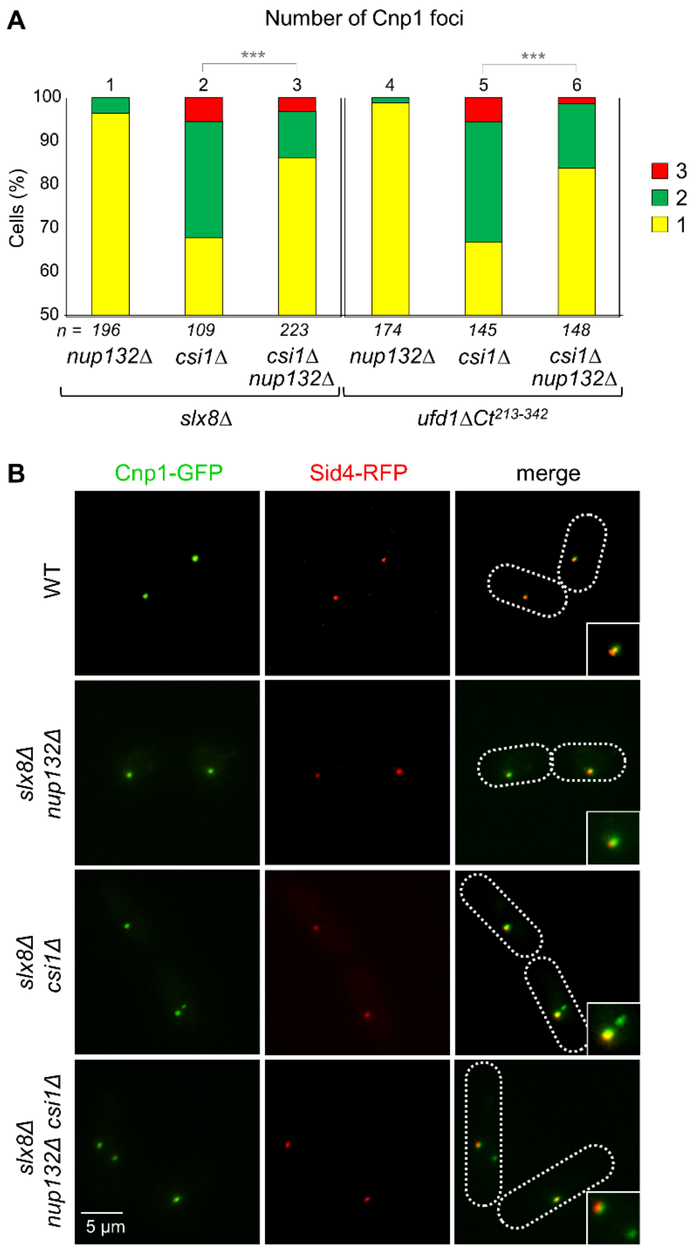

Fig. S4. SUMO-mediated enhancement of centromere clustering is not dependent on Slx8 or Ufd1. (A) Quantification of cells displaying one, two or three Cnp1 foci, based on live-cell imaging of GFP-Cnp1 (and Sid4-RFP as SPB marker). Shown are percentages based on analysis of *n* cells. Asterisks (***) denote $p \leq 0.001$ from χ2 test analysis. **(B)** Representative images from two-colour live cell imaging of GFP-Cnp1 (centromere) and Sid4-RFP (SPB). Dotted lines indicate cell boundaries.

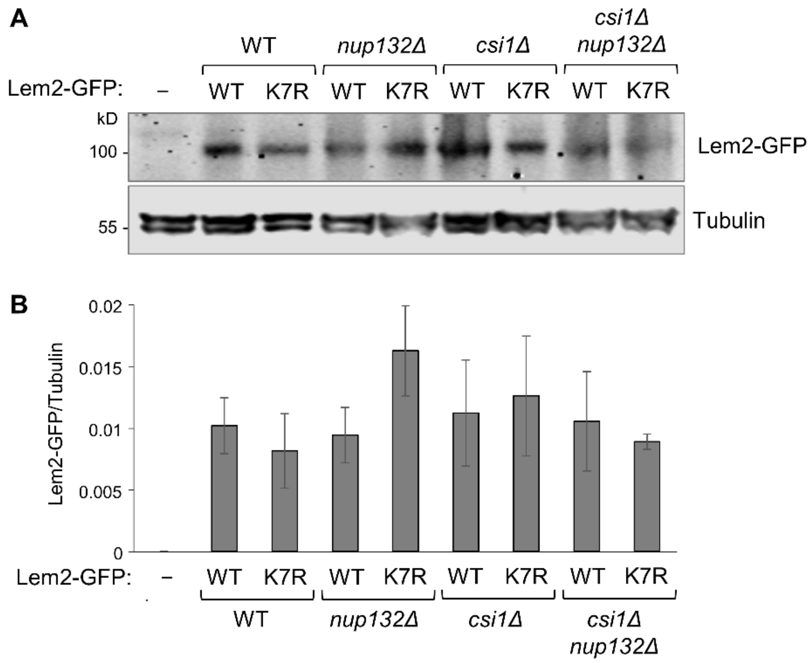

Fig. S5. Lem2 and Lem2^{K7R} show comparable stability in different mutant backgrounds. (A) Representative western blot analysis of expression levels of Lem2-GFP, or Lem2^{K7R}-GFP, in wild-type, *nup132Δ, csi1Δ, or csi1Δ nup132Δ* cells. Tubulin (anti-Tat1) serves as a loading control. **(B)** Quantitation of expression levels relative to tubulin as in (A); data plotted are the mean +/- one standard deviation from three replicates.

Journal of Cell Science • Supplementary information

Table S1. Identified ubiquitination sites.

Available for download at
https://journals.biologists.com/jcs/article-lookup/doi/10.1242/jcs.260868#supplementary-data

Table S2. Strains used in this study.

Strain	Genotype	**Figure**
3381	*h+ ade6-210 arg3-D4 his3-D1 leu1-32 ura4-D18 otr1R(dg-glu)Sph1:ade6*$^{+}$	1b
3998	*h- pli1*$^{+}$*-flag:NatR ade6-210 arg3-D4 his3-D1 leu1-32 ura4-D18 otr1R(dg-glu)Sph1:ade6*$^{+}$	1b
3999	*h- pli1*^{K3R}*-flag:NatR ade6-210 arg3-D4 his3-D1 leu1-32 ura4-D18 otr1R(dg-glu)Sph1:ade6*$^{+}$	1b
4002	*h+ nup132Δ::ura4*$^{+}$ *pli1*$^{+}$*-flag:NatR ade6-210 arg3-D4 his3-D1 leu1-32 ura4-D18 otr1R(dg-glu)Sph1:ade6*$^{+}$	1b
4003	*h- nup132Δ::ura4*$^{+}$ *pli1*^{K3R}*-flag:NatR ade6-210 arg3-D4 his3-D1 leu1-32 ura4-D18/DSE otr1R(dg-glu)Sph1:ade6*$^{+}$	1b
4371	*clr4Δ::leu2*$^{+}$ *ade6-210 leu1-32 ura4-D18/DSE imr1L(NcoI):ura4*$^{+}$	1c
737	*h- leu1-32 ura4DS/E ade6-210 his1-102 imr1L(NcoI):ura4*$^{+}$	1c, 2c, 3c, 3d, 3e, S2, S3a
4252	*pli1*$^{+}$*-flag:NatR ade6-210 arg3-D4 his3-D1 leu1-32 ura4-D18 imr1L(NcoI):ura4*$^{+}$	1c
4259	*pli1*^{K3R}*-flag:NatR ade6-210 arg3-D4 his3-D1 leu1-32 ura4-D18/DSE imr1L(NcoI):ura4*$^{+}$	1c
4365	*h+ nup132Δ::HygR ade6-210 arg3-D4 his3-D1 leu1-32 ura4-D18/DSE imr1L(NcoI):ura4*$^{+}$	1c, 2c, 3c, 3d, 3e, S2
4368	*pli1Δ::NatR ade6-210 arg3-D4 his3-D1 leu1-32 ura4-D18/DSE imr1L(NcoI):ura4*$^{+}$	1c
4330	*nup132Δ::HygR pli1*$^{+}$*-flag:NatR ade6-210 arg3-D4 his3-D1 leu1-32 ura4-D18 imr1L(NcoI):ura4*$^{+}$	1c
4370	*nup132Δ::HygR pli1*^{K3R}*-flag:NatR ade6-210 arg3-D4 his3-D1 leu1-32 ura4-D18/DSE imr1L(NcoI):ura4*$^{+}$	1c
5192	*pREP41 ade6-210 leu1-32 ura4-D18/DSE imr1L(NcoI):ura4*$^{+}$	2a
5193	*pREP41 ade6-210 leu1-32 ura4-D18/DSE imr1L(NcoI):ura4*$^{+}$	2a
5158	*pREP41-myc-his-ulp1*$^{+}$ *ade6-210 leu1-32 ura4-D18/DSE imr1L(NcoI):ura4*$^{+}$	2a
5159	*pREP41-myc-his-ulp1*$^{+}$ *ade6-210 leu1-32 ura4-D18/DSE imr1L(NcoI):ura4*$^{+}$	2a
4984	*pREP41 nup132Δ::HygR ade6-210 arg3-D4 his3-D1 leu1-32 ura4-D18/DSE imr1L(NcoI):ura4*$^{+}$	2a
4985	*pREP41 nup132Δ::HygR ade6-210 arg3-D4 his3-D1 leu1-32 ura4-D18/DSE imr1L(NcoI):ura4*$^{+}$	2a
4987	*pREP41-myc-his-ulp1*$^{+}$ *nup132Δ::HygR ade6-210 arg3-D4 his3-D1 leu1-32 ura4-D18/DSE imr1L(NcoI):ura4*$^{+}$	2a
4988	*pREP41-myc-his-ulp1*$^{+}$ *nup132Δ::HygR ade6-210 arg3-D4 his3-D1 leu1-32 ura4-D18/DSE imr1L(NcoI):ura4*$^{+}$	2a
8894	*h- pREP41 leu1-32 ura4DS/E ade6-210 his1-102 imr1L(NcoI):ura4*$^{+}$	2b
8895	*h- pREP41-myc-his-mature-pmt3*$^{+}$*leu1-32 ura4DS/E ade6-210 his1-102 imr1L(NcoI):ura4*$^{+}$	2b
8896	*h- pREP41-myc-his-mature-pmt3*^{KallR} *leu1-32 ura4DS/E ade6-210 his1-102 imr1L(NcoI):ura4*$^{+}$	2b
8897	*h+ pREP41 nup132Δ::HygR ade6-210 arg3-D4 his3-D1 leu1-32 ura4-D18/DSE imr1L(NcoI):ura4*$^{+}$	2b

8898	h+ pREP41-myc-his-mature-pmt3^{+} nup132Δ::HygR ade6-210 arg3-D4 his3-D1 leu1-32 ura4-D18/DSE imr1L(NcoI):ura4^{+}	2b
8899	h+ pREP41-myc-his-mature-pmt3^{KallR} nup132Δ::HygR ade6-210 arg3-D4 his3-D1 leu1-32 ura4-D18/DSE imr1L(NcoI):ura4^{+}	2b
9467	h- arg3Δ::GFP-nup107-his-myc-ulp1 leu1-32 ura4-D18/DSE ade6-210 imr1L(NcoI):ura4^{+}	2c
9473	nup132Δ::HygR arg3Δ::GFP-nup107-his-myc-ulp1 leu1-32 ura4-D18/DSE ade6-210 imr1L(NcoI):ura4^{+}	2c
9474	nup132Δ::HygR arg3Δ::GFP-nup107-his-myc-ulp1 leu1-32 ura4-D18/DSE ade6-210 imr1L(NcoI):ura4^{+}	2c
9478	nup132Δ::HygR arg3Δ::GFP-nup107-his-myc-ulp1 leu1-32 ura4-D18/DSE ade6-210 imr1L(NcoI):ura4^{+}	2c
9191	h- pREP41-lem2-GFP ade6-210 arg3-D4 his3-D1 leu1-32 ura4-D18 otr1R(dg-glu)Sph1:ade6^{+}	3b
9193	h- pREP41-lem2^{K7R}-GFP ade6-210 arg3-D4 his3-D1 leu1-32 ura4-D18 otr1R(dg-glu)Sph1:ade6^{+}	3b
9113	h- pREP41 ade6-210 arg3-D4 his3-D1 leu1-32 ura4-D18 otr1R(dg-glu)Sph1:ade6^{+}	3b
5820	lem2^{K7R} leu1-32 ura4-D18/DSE imr1L(NcoI):ura4^{+}	3c, 3d
5821	lem2^{K7R} leu1-32 ura4-D18/DSE imr1L(NcoI):ura4^{+}	3c
5822	nup132Δ::HygR lem2^{K7R} leu1-32 ade6^{+} ura4-D18/DSE imr1L(NcoI):ura4^{+}	3c, 3d
5182	lem2ΔN nup132Δ::HygR leu1-32 ura4-D18/DSE imr1L(NcoI):ura4^{+}	3e
5184	lem2ΔN leu1-32 ura4-D18/DSE imr1L(NcoI):ura4^{+}	3e
5513	h90 sid4^{+}-mRFP:KanR GFP-cnp1^{+}:NatR leu1-32 ura4-D18	4a, 4b, S4b
5517	h90 lem2Δ::ura4^{+} sid4^{+}-mRFP:KanR GFP-cnp1^{+}:NatR leu1-32 ura4-D18	4a, 4b
5516	h- nup132Δ::HygR sid4^{+}-mRFP:KanR GFP-cnp1^{+}:NatR leu1-32 ura4-D18	4a, 4b
6209	lem2^{K7R} sid4^{+}-mRFP:KanR GFP-cnp1^{+}:NatR leu1-32 ura4-D18	4b
6207	lem2^{K7R} nup132Δ::HygR sid4^{+}-mRFP:KanR GFP-cnp1^{+}:NatR leu1-32 ura4-D18	4b
6363	h+ csi1Δ::HygR GFP-cnp1^{+}:NatR sid4^{+}-mRFP:KanR leu1-32 ura4-D18	4c, 5c
6862	csi1Δ::HygR nup132Δ::ura4^{+} GFP-cnp1^{+}:NatR sid4^{+}-mRFP:KanR leu1-32 ura4-D18	4c
7036	pli1Δ::ura4^{+} sid4^{+}-mRFP:KanR GFP-cnp1^{+}:NatR leu1-32 ura4-D18	4c
7015	pli1Δ::ura4^{+} nup132Δ::HygR sid4^{+}-mRFP:KanR GFP-cnp1^{+}:NatR leu1-32 ura4-D18	4c
7302	h- csi1Δ::KanR pli1Δ::ura4^{+} sid4^{+}-mRFP:KanR GFP-cnp1^{+}:NatR leu1-32 ura4-D18	4c
7233	h- csi1Δ::KanR nup132Δ::HygR pli1Δ::ura4^{+} sid4^{+}-mRFP:KanR GFP-cnp1^{+}:NatR leu1-32 ura4-D18	4c
7440	h+ csi1Δ::HygR lem2^{K7R} GFP-cnp1^{+}:NatR sid4^{+}-mRFP:KanR leu1-32 ura4-D18/DSE	4c
7444	h- csi1Δ::HygR nup132Δ::HygR lem2^{K7R} GFP-cnp1^{+}:NatR sid4^{+}-mRFP:KanR leu1-32 ura4-D18/DSE	4c
7311	h90 pREP41 sid4^{+}-mRFP:KanR GFP-cnp1^{+}:NatR leu1-32 ura4-D18	4d
7315	h90 pREP41-myc-his-mature-pmt3^{+} sid4^{+}-mRFP:KanR GFP-cnp1^{+}:NatR leu1-32 ura4-D18	4d
7317	h90 pREP41-myc-his-mature-pmt3^{KallR} sid4^{+}-mRFP:KanR GFP-cnp1^{+}:NatR leu1-32 ura4-D18	4d

7313	h90 pREP41-myc-his-ulp1^{+} sid4^{+}-mRFP:KanR GFP-cnp1^{+}:NatR leu1-32 ura4-D18	4d
7319	h- pREP41 nup132Δ::HygR sid4^{+}-mRFP:KanR GFP-cnp1^{+}:NatR leu1-32 ura4-D18	4d
7323	h- pREP41-myc-his-mature-pmt3^{+} nup132Δ::HygR sid4^{+}-mRFP:KanR GFP-cnp1^{+}:NatR leu1-32 ura4-D18	4d
7325	h- pREP41-myc-his-mature-pmt3^{KallR} nup132Δ::HygR sid4^{+}-mRFP:KanR GFP-cnp1^{+}:NatR leu1-32 ura4-D18	4d
7321	h- pREP41-myc-his-ulp1^{+} nup132Δ::HygR sid4^{+}-mRFP:KanR GFP-cnp1^{+}:NatR leu1-32 ura4-D18	4d
7327	h+ pREP41 csi1Δ::HygR GFP-cnp1^{+}:NatR sid4^{+}-mRFP:KanR leu1-32 ura4-D18	4d
7331	h+ pREP41-myc-his-mature-pmt3^{+} csi1Δ::HygR GFP-cnp1^{+}:NatR sid4^{+}-mRFP:KanR leu1-32 ura4-D18	4d
7333	h+ pREP41-myc-his-mature-pmt3^{KallR} csi1Δ::HygR GFP-cnp1^{+}:NatR sid4^{+}-mRFP:KanR leu1-32 ura4-D18	4d
7329	h+ pREP41-myc-his-ulp1^{+} csi1Δ::HygR GFP-cnp1^{+}:NatR sid4^{+}-mRFP:KanR leu1-32 ura4-D18	4d
7335	pREP41 csi1Δ::HygR nup132Δ::ura4^{+} GFP-cnp1^{+}:NatR sid4^{+}-mRFP:KanR leu1-32 ura4-D18	4d
7339	pREP41-myc-his-mature-pmt3^{+} csi1Δ::HygR nup132Δ::ura4^{+} GFP-cnp1^{+}:NatR sid4^{+}-mRFP:KanR leu1-32 ura4-D18	4d
7341	pREP41-myc-his-mature-pmt3^{KallR} csi1Δ::HygR nup132Δ::ura4^{+} GFP-cnp1^{+}:NatR sid4^{+}-mRFP:KanR leu1-32 ura4-D18	4d
7337	pREP41-myc-his-ulp1^{+} csi1Δ::HygR nup132Δ::ura4^{+} GFP-cnp1^{+}:NatR sid4^{+}-mRFP:KanR leu1-32 ura4-D18	4d
8655	lem2^{+}-GFP:KanR sid4^{+}-mRFP:KanR leu1-32 ura4-D18/DSE	5a, 5b, S3b, S5
7689	h- nup132Δ::HygR lem2^{+}-GFP:KanR sid4^{+}-mRFP:KanR leu1-32 ura4-D18/DSE	5a, 5b, S3b, S5
7691	h+ csi1Δ::HygR lem2^{+}-GFP:KanR sid4^{+}-mRFP:KanR leu1-32 ura4-D18/DSE	5a, 5b, S5
7693	nup132Δ::HygR csi1Δ::HygR lem2^{+}-GFP:KanR sid4^{+}-mRFP:KanR leu1-32 ura4-D18/DSE	5a, 5b. S5
8639	lem2^{K7R}-GFP:KanR sid4^{+}-mRFP:KanR leu1-32 ura4-D18/DSE	5b, S3b, S5
7695	h- nup132Δ::HygR lem2^{K7R}-GFP:KanR sid4^{+}-mRFP:KanR leu1-32 ura4-D18/DSE	5b, S3b, S5
7744	csi1Δ::HygR lem2^{K7R}-GFP:KanR sid4^{+}-mRFP:KanR leu1-32 ura4-D18/DSE	5b, S5
7743	nup132Δ::HygR csi1Δ::HygR lem2^{K7R}-GFP:KanR sid4^{+}-mRFP:KanR leu1-32 ura4-D18/DSE	5b, S5
8717	bqt4Δ::NatR GFP-cnp1^{+}:NatR sid4^{+}-mRFP:KanR ura4-D18/DSE	5c
8714	csi1Δ::HygR bqt4Δ::NatR GFP-cnp1^{+}:NatR sid4^{+}-mRFP:KanR ura4-D18/DSE	5c
4	h- wild type S. pombe	S1
5919	h+ nup132Δ::HygR lem2^{+}-GFP:KanR ade6-210 leu1-32 ura4-D18/DSE imr1L(NcoI):ura4^{+}	S3a
5908	h+ nup132Δ::HygR lem2^{K7R}-GFP:KanR leu1-32 ura4-D18/DSE imr1L(NcoI):ura4^{+}	S3a
5882	h+ lem2^{+}-GFP:KanR leu1-32 ura4DS/E ade6-210 his1-102 imr1L(NcoI):ura4^{+}	S3a
5884	h- lem2^{K7R}-GFP:KanR leu1-32 ura4-D18/DSE imr1L(NcoI):ura4^{+}	S3a

7679	*slx8Δ::KanR nup132Δ::HygR GFP-cnp1*$^{+}$*:NatR sid4*$^{+}$*-mRFP:KanR leu1-32 ura4-D18/DSE*	S4
8000	*slx8Δ::KanR csi1Δ::HygR sid4*$^{+}$*-mRFP:KanR GFP-cnp1*$^{+}$*:NatR ura4-D18 leu1-32*	S4
7745	*csi1Δ::HygR nup132Δ::ura4*$^{+}$ *slx8Δ::KanR GFP-cnp1*$^{+}$*:NatR sid4*$^{+}$*-mRFP:KanR leu1-32 ura4-D18/DSE*	S4
8282	*h+ ufd1ΔCt*$^{213\text{-}342}$*:HygR nup132Δ::ura4*$^{+}$ *sid4*$^{+}$*-mRFP:KanR GFP-cnp1*$^{+}$*:NatR leu1-32 ura4-D18/DSE*	S4
8208	*h- csi1Δ::KanR ufd1ΔCt*$^{213\text{-}342}$*:HygR sid4*$^{+}$*-mRFP:KanR GFP-cnp1*$^{+}$*:NatR leu1-32 ura4-D18/DSE*	S4
8291	*ufd1ΔCt*$^{213\text{-}342}$*:HygR csi1Δ::KanR nup132Δ::ura4*$^{+}$ *sid4*$^{+}$*-mRFP:KanR GFP-cnp1*$^{+}$*:NatR leu1-32 ura4-D18/DSE*	S4
7018	*pli1Δ::ura4*$^{+}$ *nup132Δ::HygR sid4*$^{+}$*-mRFP:KanR leu1-32 ura4-D18*	S5

Table S3. Plasmids used in this study.

Plasmid	Source	Figure
pREP41-myc-his	[1]Craven et al., 1998	2a, 2b, 3b, 4d
pREP41-myc-his-ulp1^{+}	This study	2a, 4d
pREP41-myc-his-mature-pmt3^{+}	This study	2b, 4d
pREP41-myc-his-mature-pmt3^{KallR}	This study	2b, 4d
pREP41-lem2-GFP	This study	3b
pREP41-lem2^{K7R}***-GFP***	This study	3b

[1]Craven, R.A., D.J. Griffiths, K.S. Sheldrick, R.E. Randall, I.M. Hagan, and A.M. Carr. 1998. Vectors for the expression of tagged proteins in Schizosaccharomyces pombe. Gene. 221:59-68.
