# Supplementary material for: SUMOylation regulates Lem2 function in centromere clustering and silencing
Source: J Cell Sci. 2023 Dec 1;136(23):jcs260868. doi: 10.1242/jcs.260868 (PMC10730020; doi:10.1242/jcs.260868)
Supplement: Supplementary file 1 [file joces-136-260868_review_history.pdf]

Journal of Cell Science

SUMOylation regulates Lem2 function in centromere clustering and silencing

Joanna Strachan, Orsolya Leidecker, Christos Spanos, Clementine Le Coz, Elliott Chapman, Ana Arsenijevic, Haidao Zhang, Ning Zhao, Steven H Spoel and Elizabeth Helen Bayne
DOI: 10.1242/jcs.260868

Editor: David Glover

Review timeline
Original submission: 2 December 2022
Editorial decision: 9 February 2023
First revision received: 11 October 2023
Accepted: 7 November 2023

Original submission

First decision letter

MS ID#: JOCES/2022/260868

MS TITLE: SUMOylation regulates Lem2 function in centromere clustering and silencing

AUTHORS: Joanna Strachan, Orsolya Leidecker, Christos Spanos, Clementine Le Coz, Elliott Chapman, Ana Arsenijevic, Haidao Zhang, Ning Zhao, and Elizabeth Helen Bayne
ARTICLE TYPE: Research Article

Thank you for your recent letter.

I’m quite happy for you to revise your paper in which case I would send it out to the same referees plus one new referee. Please go ahead and make the revisions, resubmit and please send me some more suggestions of potential referees. We can then re-evaluate.

To submit a revision, please go to: https://submit-jcs.biologists.org

Go to the Author Area and click on the 'Submit a Revision' link.

Best wishes,
David Glover

Editor

Journal of Cell Science
The Company of Biologists Ltd
Bidder Building
Station Road
Histon, Cambridge, CB24 9LF
Phone:44 (0)1223 632867
Fax:44 (0)1223 632869
jcs@biologists.com

The information contained in this message and any attachment is confidential, legally privileged and is intended for the addressee only. Any dissemination, distribution, copying, disclosure or use

© 2023. Published by The Company of Biologists under the terms of the Creative Commons Attribution License (https://creativecommons.org/licenses/by/4.0/).

of this message/attachment or its contents is strictly prohibited and may be unlawful. No contract is intended or implied, unless confirmed by hard copy. If you have received this message in error, please inform the sender and delete it from your mailbox or any other storage mechanism.

The Company Of Biologists Ltd cannot accept liability for any statements made which are clearly the senders' own and not expressly made on behalf of the Company of Biologists Limited or one of their agents.

Reviewer 1

Advance summary and potential significance to field

In this manuscript, the lab of Elizabeth Bayne has investigated how the spatial regulation of SUMOylation contributes to centromere biology, including centromeric silencing and centromere clustering at the spindle pole body (SPB). The data established that the delocalization of the SUMO protease Ulp1 from the nuclear periphery (i.e in the nup132 mutant) results in opposite effects on centromere, leading to defect in centromeric silencing while improving centromere clustering, especially in the absence of Csi1. Mechanistically, the authors established that those effects are caused by an hyper-SUMOylation at the nuclear periphery rather than a defect in the SUMO E3 ligase Pli1. Furthermore, the authors established that the centromere defects are in part caused by a deregulation of the SUMOylation of the inner nuclear membrane protein Lem2. Cell biology experiments further support the conclusion that SUMOylated forms of Lem2 act as a regulatory switch to modulate Lem2 interactions with unknown partners in order to balance Lem2 function in centromeric silencing versus centromere clustering. Overall, the manuscript is very well-written, the logic is easy to follow, the genetic experiments are robust and elegant. The discovery that Lem2 has distinct functions in promoting centromeric silencing and centromere clustering and that SUMOylation may act as a regulatory switch to balance these functions provide a clear advance in the field. In addition, an atlas of ubiquitinated proteins in S. pombe is provided which will be of most interest to broad scientific community.

Comments for the author

Major points
There are two caveats in this manuscript that the authors should try to address to make their conclusions stronger.
1. There is no direct evidence that Lem2 is hyperSUMOylated in the nup132 mutant and that the mutated form Lem2-K7R is defective for SUMOylation. Most of the conclusions are based on the assumption that mutating the 7 acceptor lysines in the N-terminal part results in defective SUMOylation, a point that is not demonstrated in the manuscript. Therefore, the authors cannot exclude that the Lem2-7KR mutant is defective for protein-protein interactions, independently of SUMoylation, to balance its function in centromeric silencing and centromere clustering. A blot showing Lem2 SUMOylation level in the nup132 mutant and in the lem2-7KR mutant is necessary to strengthen the conclusions. It seems that the authors have all the tools in hand to address this point (Lem2-GFP, Myc-His-SUMO).
2. The conclusion that the centromeric silencing defect of the nup132 mutant is caused by an hyperSUMOylation at the nuclear periphery is built on indirect genetic evidences (rescue by Ulp1 overexpression and Pmt3-KallR overexpression) that do not lead to the restoration of normal SUMOylation level specifically at the nuclear periphery. Given the broad effect of the nup132 mutant and SUMOylation on macromolecular transport, more direct evidences would strengthen the conclusion. Maybe the authors could try to fuse the catalytic domain of Ulp1 to a nucleoporin to force Ulp1 localisation at the nuclear periphery in the nup132 mutant. A similar strategy was successfully implemented in budding yeast (see PMID: 12471376 and PMID: 24074957).

Minor points
3. Page 7, second paragraph, third line “as a result of either Pli1 stabilisation” should be “destabilisation” ?
4. Do the authors have any idea why the centromeric silencing defect of the nup132 mutant is only observed on rich medium and not on minimal medium? (page 10, Figure S2)

© 2023. Published by The Company of Biologists under the terms of the Creative Commons Attribution License (https://creativecommons.org/licenses/by/4.0/).

5. How is the nuclear shape in the slx8 null mutant ? (Figure S4). This strain is known to be extremely sick. A couple of representative cell images will be appropriated.
6. the lack of polySUMOylation leads to a slight defect in centromere clustering (bar 3 on Figure 3D). Is this effect statistically different from WT ?

Reviewer 2

Advance summary and potential significance to field

In this manuscript Strachan et al have examined the basis of the centromere defects in S. pombe cells lacking the nucleoporin, Nup132. They find that contrary to previous suggestions, the defects associated with this mutant result from 'hypersumoylation' and they identify Lem2 as an important sumoylation substrate in this context. The authors also show that destabilisation of the E3 ligase, Pli1 does not cause the centromeric silencing defects associated with nup132 cells. They also show that expression of a mutant Lem2 (Lem2K7R) can suppress the centromeric transcriptional silencing defects of a nup132 mutant.
In addition, the authors present a global analysis of ubiquitination sites in S. pombe which is much more extensive than that previously reported by the Gould lab (Beckley et al 2015).

Comments for the author

The problems/limitations with the study relate mainly to the analyses of sumoylation on centromere clustering and Lem2 localisation (Figs 3 and 4). The major issue here is that the changes observed in the various mutant backgrounds are subtle at best. For example loss of Csi1 results in only a very modest (~35%) reduction in cells with a single Cnp1/CENP-A signal and deletion of nup132 in this background only partially suppresses this subtle defect. Indeed many of the conclusions from Figs 3 and 4 are based on very small percentage changes and are not convincing. Another major issue is that authors do not directly measure changes in sumoylation (global and poly) that result from their various genetic manipulations (e.g. overexpression of Ulp1 and Pmt3-KallR).
There is also no formal demonstration that the K7R mutant abolishes or even influences the sumoylation of Lem2. There are 24 lysine residues in the N-terminal region of Lem2 and so sumoylation of the K7R mutant at alternative sites is still possible. Overall there is no direct evidence to support the conclusion that phenotypes of this mutant result from loss of sumoylation. Given these considerations I do not think this study is suitable for publication.

Other points
Fig 1B Pli1 levels should be quantified in the different backgrounds.
Page 10. The term 'hypersumoylation' needs to be defined. Do the authors mean an increase in the global level of sumo (Pmt3), an increase in the total number of sumoylated proteins or an increase in the level poly (high mol wt) sumoylation? (The data here seem to suggest that it is a reduction the levels of in poly sumoylation that can suppress the nup132 phenotypes). The affect of nup132 deletion and overexpression of Ulp1 and Pmt3-KallR on global levels of sumo and high mol wt sumoylation should be shown.
The authors should consider expressing a Lem2-sumo fusion (similar to Ross et al 2002. DOI: 10.1016/s1097-2765(02)00682-2). If their model is correct then over expression of this fusion may mimic the phenotypes associated with loss of nup132 The authors suggest that there is a small (~5%) reduction in SPB localisation of Lem2-K7R compared to wild type Lem2 in the absence of Csi1. Is this statistically significant? Similarly, is the difference between wt Lem2 and K7R localisation in the csi1 nup132 background statistically significant? Given the very small differences in localisation and clustering, it would also be important to quantify the levels of Lem2 wt and K7R in all genetic backgrounds under study.
Both the Introduction and Discussion section seem to be overly long. Could these sections be shortened?

First revision

© 2023. Published by The Company of Biologists under the terms of the Creative Commons Attribution License (https://creativecommons.org/licenses/by/4.0/).

Author response to reviewers' comments

Response to Reviewers - JOCES/2022/260868

Reviewer 1 Advance Summary and Potential Significance to Field...
In this manuscript, the lab of Elizabeth Bayne has investigated how the spatial regulation of SUMOylation contributes to centromere biology, including centromeric silencing and centromere clustering at the spindle pole body (SPB). The data established that the delocalization of the SUMO protease Ulp1 from the nuclear periphery (i.e in the nup132 mutant) results in opposite effects on centromere, leading to defect in centromeric silencing while improving centromere clustering, especially in the absence of Csi1.
Mechanistically, the authors established that those effects are caused by an hyper- SUMOylation at the nuclear periphery rather than a defect in the SUMO E3 ligase Pli1. Furthermore, the authors established that the centromere defects are in part caused by a deregulation of the SUMOylation of the inner nuclear membrane protein Lem2. Cell biology experiments further support the conclusion that SUMOylated forms of Lem2 act as a regulatory switch to modulate Lem2 interactions with unknown partners in order to balance Lem2 function in centromeric silencing versus centromere clustering. Overall, the manuscript is very well-written, the logic is easy to follow, the genetic experiments are robust and elegant. The discovery that Lem2 has distinct functions in promoting centromeric silencing and centromere clustering and that SUMOylation may act as a regulatory switch to balance these functions provide a clear advance in the field. In addition, an atlas of ubiquitinated proteins in S. pombe is provided which will be of most interest to broad scientific community.

We thank the reviewer for their very positive comments including noting that our genetic experiments are robust and elegant, and that our findings represent a clear advance in the field.

Reviewer 1 Comments for the Author...
Major points
There are two caveats in this manuscript that the authors should try to address to make their conclusions stronger.
1. There is no direct evidence that Lem2 is hyperSUMOylated in the nup132 mutant and that the mutated form Lem2-K7R is defective for SUMOylation. Most of the conclusions are based on the assumption that mutating the 7 acceptor lysines in the N-terminal part results in defective SUMOylation, a point that is not demonstrated in the manuscript. Therefore, the authors cannot exclude that the Lem2-7KR mutant is defective for protein- protein interactions, independently of SUMoylation, to balance its function in centromeric silencing and centromere clustering. A blot showing Lem2 SUMOylation level in the nup132 mutant and in the lem2-7KR mutant is necessary to strengthen the conclusions. It seems that the authors have all the tools in hand to address this point (Lem2-GFP, Myc- His-SUMO).

We acknowledge this point and indeed we had previously attempted to detect Lem2 SUMOylation by western blot, but without success. However, after further optimisation of the approach we are pleased to say that we have now been able to observe SUMOylation of wild-type Lem2 by IP-western. Moreover, we have confirmed that this SUMOylation is indeed lost in the Lem2^{K7R} mutant as expected, confirming that the phenotypes can likely be attributed to loss of SUMOylation. The new blots showing this are presented in Figure 3B.

Unexpectedly, by this same method we could not detect SUMOylated Lem2 in the *nup132Δ* background. Taken at face value, this result is the opposite of the hyper- SUMOylation of Lem2 implied by all of our genetic data. However, we suspect that this is most likely an artefact arising from hyper-SUMOylated species being rendered insoluble due to aggregation, and therefore undetectable by IP, as has been reported previously for other hyper-SUMOylated proteins (e.g. O’Rourke et al, PMID 23871671; Renner et al, PMID 20188669; this phenomenon is particularly associated with aggregation-prone proteins, which we suspect Lem2 is, since human LEM2 has been shown to phase separate). It is also possible that highly SUMOylated species may be turned over and/or deSUMOylated more rapidly. Since we have been unable to definitively confirm the SUMOylation status of Lem2 in *nup132Δ* cells, we have modified the text slightly to avoid the

© 2023. Published by The Company of Biologists under the terms of the Creative Commons Attribution License (https://creativecommons.org/licenses/by/4.0/).

specific claim that Lem2 is hyper-SUMOylated in this background. However, this does not affect our overall conclusions; regardless of the exact nature of the change, the difference observed by IP does nevertheless provide direct evidence that Lem2 SUMOylation status is altered in *nup132Δ* cells. Together with our genetic data indicating that blocking Lem2 SUMOylation ($Lem2^{K7R}$) helps suppress effects of *nup132* deletion, this further supports our conclusion that changes in Lem2 SUMOylation status contribute to altered regulation of centromere clustering and silencing in this background.

2. The conclusion that the centromeric silencing defect of the nup132 mutant is caused by hyperSUMOylation at the nuclear periphery is built on indirect genetic evidences (rescue by Ulp1 overexpression and Pmt3-KallR overexpression) that do not lead to the restoration of normal SUMOylation level specifically at the nuclear periphery. Given the broad effect of the nup132 mutant and SUMOylation on macromolecular transport, more direct evidences would strengthen the conclusion. Maybe the authors could try to fuse the catalytic domain of Ulp1 to a nucleoporin to force Ulp1 localisation at the nuclear periphery in the nup132 mutant. A similar strategy was successfully implemented in budding yeast (see PMID: 12471376 and PMID: 24074957).

We thank the reviewer for this constructive suggestion. We have now done the experiment described of fusing Ulp1 to the nucleoporin Nup107, and indeed this does lead to a rescue in centromeric silencing and TBZ sensitivity in *nup132Δ* cells, further supporting our conclusion that the defect is a result of hyper-SUMOylation at the nuclear periphery. The rescue is not complete, but this is unsurprising since the tethering of Ulp1 is an artificial scenario that likely also does not result in fully wild-type SUMOylation levels. The new data are shown in Figure 2C.

Minor points

3. Page 7, second paragraph, third line “as a result of either Pli1 stabilisation” should be “destabilisation” ?

This has been corrected.

4. Do the authors have any idea why the centromeric silencing defect of the nup132 mutant is only observed on rich medium and not on minimal medium? (page 10, Figure S2)

No - this phenomenon has been noted before (Tange et al, PMID: 27334362) and why it comes about is an interesting question, but we still do not have an answer at present.

5. How is the nuclear shape in the slx8 null mutant ? (Figure S4). This strain is known to be extremely sick. A couple of representative cell images will be appropriated.

As reported previously, deletion of $nup132^{+}$ rescues sickness in *slx8* mutant cells (Nie & Boddy, PMID: 26221037). We have added representative images as requested to Figure S4.

6. the lack of polySUMOylation leads to a slight defect in centromere clustering (bar 3 on Figure 3D). Is this effect statistically different from WT ?

Yes, this difference is statistically significant, consistent with polySUMOylation contributing to centromere clustering. We have now indicated this in the figure and commented on it in the text. (p.14: “Interestingly, even in wild-type cells we found that over-expression of $Pmt3^{KallR}$, which suppresses polySUMOylation, resulted in a small but significant defect in centromere clustering, consistent with a role for polySUMOylation in promoting clustering.”)

Reviewer 2 Advance Summary and Potential Significance to Field...

In this manuscript Strachan et al have examined the basis of the centromere defects in S. pombe cells lacking the nucleoporin, Nup132. They find that contrary to previous suggestions, the defects associated with this mutant result from ‘hypersumoylation’ and they identify Lem2 as an important sumoylation substrate in this context. The authors also show that destabilisation of the E3 ligase, Pli1 does not cause the centromeric silencing defects associated with nup132 cells. They also show that expression of a mutant Lem2 (Lem2K7R) can suppress the centromeric transcriptional silencing defects of a nup132 mutant. In addition, the authors present a global analysis of ubiquitination sites in S. pombe which is much more extensive than that previously reported by the Gould lab (Beckley et al 2015).

© 2023. Published by The Company of Biologists under the terms of the Creative Commons Attribution License (https://creativecommons.org/licenses/by/4.0/).

We thank the reviewer for their nice summary of the advances represented by our findings.

Reviewer 2 Comments for the Author...
The problems/limitations with the study relate mainly to the analyses of sumoylation on centromere clustering and Lem2 localisation (Figs 3 and 4). The major issue here is that the changes observed in the various mutant backgrounds are subtle at best. For example loss of Csi1 results in only a very modest (~35%) reduction in cells with a single Cnp1/CENP-A signal and deletion of nup132 in this background only partially suppresses this subtle defect. Indeed, many of the conclusions from Figs 3 and 4 are based on very small percentage changes and are not convincing.

To someone outside the immediate field these may seem like modest changes, but our observations for *csi1Δ* are in line with previous studies (e.g. the ~35% reduction in cells with a single Cnp1 focus shown here is comparable to the ~40% reduction reported in Barrales et al, PMID: 26744419), and in fact these represent relatively severe defects compared to those associated with deletion of other factors shown to contribute to clustering, such as Lem2 (~15% reduction in our hands, and ~10% in Barrales et al.). The changes we report are both highly reproducible, and entirely consistent with the scale of changes typically seen in the context of this system; we are therefore confident that they are functionally meaningful.

Another major issue is that authors do not directly measure changes in sumoylation (global and poly) that result from their various genetic manipulations (e.g. overexpression of Ulp1 and Pmt3-KallR). There is also no formal demonstration that the K7R mutant abolishes or even influences the sumoylation of Lem2. There are 24 lysine residues in the N-terminal region of Lem2 and so sumoylation of the K7R mutant at alternative sites is still possible. Overall there is no direct evidence to support the conclusion that phenotypes of this mutant result from loss of sumoylation. Given these considerations I do not think this study is suitable for publication.

In our view, analysing global levels of SUMOylation is not particularly informative given that what we are interested in are spatially localised effects. For example, published data for *nup132Δ* cells indicate a global reduction in SUMOylation (Nie & Boddy, PMID: 26221037) that masks simultaneous hyper-SUMOylation at the nuclear periphery (Kramarz et al, PMID: 33159083).
However, the point in relation to Lem2 specifically is well taken, and as described above we have now succeeded in detecting SUMOylated Lem2 by western blot, and have confirmed that this SUMOylation is lost in the K7R mutant. The new data, shown in Figure 3B, provide the important confirmation that the phenotypes of this mutant can indeed be attributed to loss of SUMOylation.

Other points
Fig 1B Pli1 levels should be quantified in the different backgrounds.
We have added quantification to the figure.

Page 10. The term ‘hypersumoylation’ needs to be defined. Do the authors mean an increase in the global level of sumo (Pmt3), an increase in the total number of sumoylated proteins or an increase in the level poly (high mol wt) sumoylation? (The data here seem to suggest that it is a reduction the levels of in poly sumoylation that can suppress the nup132 phenotypes).
We have clarified this in the text (p.10: “…suggesting that increased poly-SUMOylation in particular contributes to the defect”)

The affect of nup132 deletion and overexpression of Ulp1 and Pmt3-KallR on global levels of sumo and high mol wt sumoylation should be shown.
It has been shown previously that deletion of *nup132*$^{+}$ results in a reduction in global SUMOylation (Nie & Boddy, PMID: 26221037). However, as noted above, we would argue that this and other global level effects are not particularly relevant or informative, as they say nothing about the spatially localised effects that are the focus of this study (in the case of *nup132*$^{+}$ deletion, hyper-SUMOylation at the nuclear periphery; Kramarz et al, PMID: 33159083).

The authors should consider expressing a Lem2-sumo fusion (similar to Ross et al 2002. DOI: 10.1016/s1097-2765(02)00682-2). If their model is correct then over expression of this fusion may mimic the phenotypes associated with loss of nup132

© 2023. Published by The Company of Biologists under the terms of the Creative Commons Attribution License (https://creativecommons.org/licenses/by/4.0/).

We have previously considered trying this experiment, however in our view any results would likely be difficult to interpret since the phenotypes associated with loss of Nup132 are similar to those associated with loss of function of Lem2. This is a particular problem since an N-terminal SUMO fusion would be most appropriate given that reported Lem2 SUMOylation sites are near the N-terminus; however, the N-terminal region of Lem2 has also been shown to mediate Lem2's association at centromeres. For example, based on our model, expression of a SUMO-Lem2 fusion would be predicted to result in defects in centromere silencing mimicking loss of Nup132, but as a similar phenotype is seen upon deletion of Lem2, we would not be able to rule out that this was the result of a non- specific loss of function caused by the fusion, rather than a specific effect of the presence of SUMO. We therefore consider that this experiment is unlikely to be helpful in strengthening our argument.

The authors suggest that there is a small (~5%) reduction in SPB localisation of Lem2-K7R compared to wild type Lem2 in the absence of Csi1. Is this statistically significant? Similarly, is the difference between wt Lem2 and K7R localisation in the csi1 nup132 background statistically significant? Given the very small differences in localisation and clustering, it would also be important to quantify the levels of Lem2 wt and K7R in all genetic backgrounds under study.
The differences mentioned are not statistically significant - we have added acknowledgment of this in the text. We have quantified the levels of wild-type Lem2 and Lem2^{K7R} in the different genetic backgrounds and found them to be comparable - the data are shown in new Figure S5.

Both the Introduction and Discussion section seem to be overly long. Could these sections be shortened?
We acknowledge that the introduction and discussion are quite long - this is because it is a complex topic with several different areas to introduce/discuss. We believe that all of the content is relevant, concise and helpful to the reader (and indeed reviewer 1 commented that the manuscript is well written), however we can reduce it if required.

Second decision letter

MS ID#: JOCES/2022/260868

MS TITLE: SUMOylation regulates Lem2 function in centromere clustering and silencing

AUTHORS: Joanna Strachan, Orsolya Leidecker, Christos Spanos, Clementine Le Coz, Elliott Chapman, Ana Arsenijevic, Haidao Zhang, Ning Zhao, Steven H Spoel, and Elizabeth Helen Bayne
ARTICLE TYPE: Research Article

I am happy to tell you that your manuscript has been accepted for publication in Journal of Cell Science, pending standard ethics checks.

Reviewer 1

Advance summary and potential significance to field

In this manuscript, the lab of Elizabeth Bayne has investigated how the spatial regulation of SUMOylation contributes to centromere biology, including centromeric silencing and centromere clustering at the spindle pole body (SPB).
The data established that the delocalization of the SUMO protease Ulp1 from the nuclear periphery (i.e in the nup132 mutant) results in opposite effects on centromere, leading to defect in centromeric silencing while improving centromere clustering, especially in the absence of Csi1. Mechanistically, the authors established that those effects are caused by an hyper SUMOylation at the nuclear periphery rather than a defect in the SUMO E3 ligase Pli1. Furthermore the authors established that the centromere defects are in part caused by a deregulation of the SUMOylation of the inner nuclear membrane protein Lem2. Cell biology experiments further support the conclusion that SUMOylated forms of Lem2 act as a regulatory switch to modulate Lem2 interactions with

© 2023. Published by The Company of Biologists under the terms of the Creative Commons Attribution License (https://creativecommons.org/licenses/by/4.0/).

unknown partners in order to balance Lem2 function in centromeric silencing versus centromere clustering. Overall, the manuscript is very well-written, the logic is easy to follow, the genetic experiments are robust and elegant. The discovery that Lem2 has distinct functions in promoting centromeric silencing and centromere clustering and that SUMOylation may act as a regulatory switch to balance these functions provide a clear advance in the field. In addition, an atlas of ubiquitinated proteins in S. pombe is provided which will be of most interest to broad scientific community

Comments for the author

In this revised form of the article, the new results provide a satisfactory response to the criticisms raised. The data showing that Lem2 is indeed SUMOylated and that the Lem2-K7R mutant impairs this SUMOylation are convincing. The authors did also their best to restore Ulp1 localisation at the nuclear periphery in the nup132 mutant.
minor point:
in the references section, kramarz et al 2021 (Kramarz, K., Saada, A. A. and Lambert, S. A. E. (2021). The Analysis of Recombination-
Dependent Processing of Blocked Replication Forks by Bidimensional Gel Electrophoresis. Methods Mol Biol 2153, 365-381) is unlikely the correct reference. I guess, it should be PMID: 33159083.

<u>Reviewer 2</u>

Advance summary and potential significance to field

In this manuscript Strachan et al have examined the basis of the centromere defects in S. pombe cells lacking the nucleoporin, Nup132. They find that contrary to previous suggestions, the defects associated with this mutant result from ‘hypersumoylation’ and they identify Lem2 as an important sumoylation substrate in this context. The authors also show that destabilisation of the E3 ligase, Pli1 does not cause the centromeric silencing defects associated with nup132 cells. They also show that expression of a mutant Lem2 (Lem2K7R) can suppress the centromeric transcriptional silencing defects of a nup132 mutant.
In addition, the authors present a global analysis of ubiquitination sites in S. pombe which is much more extensive than that previously reported by the Gould lab (Beckley et al 2015). The manuscript also contains a comprehensive analysis of ubiquitin sites. This identified ~1800 ubiquitylated proteins and >5000 sites. This will be a very useful resource for the community.

Comments for the author

The authors have satisfactorily addressed this reviewer's issues. Importantly they have demonstrated that Lem2 is sumoylated and that this is lost in the K7R mutant. This additional experiment strengthens the manuscript significantly. The authors have also addressed the other main issue which related to the analyses of centromere clustering. I note that my original concern was not shared by the other reviewer and so I am happy to accept the authors' explanation/rebuttal of the point I raised. In summary, I believe that this manuscript is now suitable for publication in Journal of Cell Science.

© 2023. Published by The Company of Biologists under the terms of the Creative Commons Attribution License (https://creativecommons.org/licenses/by/4.0/).
